# Is There Room for Second-Generation Antipsychotics in the Pharmacotherapy of Panic Disorder? A Systematic Review Based on PRISMA Guidelines

**DOI:** 10.3390/ijms17040551

**Published:** 2016-04-13

**Authors:** Giampaolo Perna, Alciati Alessandra, Balletta Raffaele, Mingotto Elisa, Diaferia Giuseppina, Cavedini Paolo, Nobile Maria, Caldirola Daniela

**Affiliations:** 1 Department of Clinical Neurosciences, Hermanas Hospitalarias, Villa San Benedetto Menni Hospital, FoRiPsi, via Roma 16, Albese con Cassano, 22032 Como, Italy; alessandra.alciati@gmail.com (A.A.); ballettalello@gmail.com (B.R.); elisamingotto@gmail.com (M.E.); g.diaferia@ospedaliere.it (D.G.); p.cavedini@paolocavedini.com (C.P.); maria.nobile4@gmail.com (N.M.); caldiroladaniela@gmail.com (C.D.); 2 Department of Psychiatry and Neuropsychology, Faculty of Health, Medicine and Life Sciences, Maastricht University, 6200 Maastricht, The Netherlands; 3 Department of Psychiatry and Behavioral Sciences, Leonard Miller School of Medicine, Miami University, Miami, FL 33136, USA; 4 Child Psychopathology Unit, Scientific Institute, IRCCS Eugenio Medea, Bosisio Parini, 23842 Lecco, Italy

**Keywords:** panic disorder, second-generation antipsychotics, quetiapine, risperidone, ziprasidone, bipolar disorder

## Abstract

A role for second-generation antipsychotics (SGAs) in the treatment of panic disorders (PD) has been proposed, but the actual usefulness of SGAs in this disorder is unclear. According to the PRISMA guidelines, we undertook an updated systematic review of all of the studies that have examined, in randomized controlled trials, the efficacy and tolerability of SGAs (as either monotherapy or augmentation) in the treatment of PD, with or without other comorbid psychiatric disorders. Studies until 31 December 2015 were identified through PubMed, PsycINFO, Embase, Cochrane Library and Clinical trials.gov. Among 210 studies, five were included (two involving patients with a principal diagnosis of PD and three involving patients with bipolar disorder with comorbid PD or generalized anxiety disorder). All were eight-week trials and involved treatments with quetiapine extended release, risperidone and ziprasidone. Overall, a general lack of efficacy of SGAs on panic symptoms was observed. Some preliminary indications of the antipanic effectiveness of risperidone are insufficient to support its use in PD, primarily due to major limitations of the study. However, several methodological limitations may have negatively affected all of these studies, decreasing the validity of the results and making it difficult to draw reliable conclusions. Except for ziprasidone, SGAs were well tolerated in these short-term trials.

## 1. Introduction

Panic disorder (PD) is a highly prevalent (lifetime prevalence rate of 3%–4%), debilitating psychiatric disorder [1]. PD results from the interplay of unexpected panic attacks (PAs) (*i.e.*, the core symptom of the disorder) and other symptoms following the occurrence of PAs, *i.e.*, anticipatory anxiety and maladaptive changes in behavior related to PAs. Most subjects with PD fear or avoid multiple situations in which PAs can occur (agoraphobia) [2].

Several medications are effective for PD, including selective serotonin reuptake inhibitors (SSRIs), serotonin-norepinephrine reuptake inhibitors (SNRIs), tricyclic antidepressants (TCAs) and benzodiazepines. Among these, SSRIs, such as paroxetine, sertraline, fluoxetine and citalopram, and SNRIs, such as venlafaxine, are considered to be first-line treatment agents because of their efficacy and favorable side effect (SE) profile [3,4].

Despite these treatment options, in short-term clinical trials, 17%–64% of participants with PD did not respond adequately to pharmacotherapy and continued to have PAs and/or avoidance symptoms [5]. Overall, approximately 20%–40% of patients did not achieve full remission with the recommended drugs. A similar percentage of patients did not improve with cognitive behavioral therapy (CBT), and combining CBT with pharmacotherapy has not sufficiently filled this gap. Finally, the rate of relapses within six months of drug discontinuation is 25%–50%, the rate of residual panic-phobic symptoms is up to 50%, and up to 30% of patients still have a full-blown disorder after 3–6 years [3,6].

From a clinical perspective, there is still a strong unmet need for more effective pharmacological treatments for PD. Disappointingly, in the last few years, the preclinical and clinical investigation of alternative novel mechanism-based antipanic drugs has made little progress, and new medications for PD are far from being implemented in clinical use [7]. On the other hand, some existing medications already approved for other psychiatric disorders having different pharmacodynamic profiles compared to standard drugs for PD have been investigated as monotherapy or adjunctive treatments to recommended antipanic therapies [3]. Among these, several studies focused on second-generation antipsychotics (SGAs). Even if the results are mixed, some preclinical and clinical studies have suggested intrinsic anxiolytic properties of these compounds, not attributable to their antipsychotic effects. In rats, risperidone, olanzapine and clozapine modulated fear conditioning and defensive behaviors against threats [8,9,10], which are the processes implicated in several anxiety conditions of humans, such as conditioned anxiety and phobic behaviors of patients with PD [11,12,13,14]. These properties of SGAs in animal models may be partly explained by their ability to modulate the dopaminergic system in prefrontal cortex and limbic regions, which are the areas involved in fear conditioning processes [10,15,16]. The SGA-induced blockade of 5-HT (serotonin) 2A receptors and activation of non-5HT 2A receptors, the enhancing of 5-HT release through blockade of α2-adrenergic receptors on 5-HT terminals and the modulation of the noradrenergic system with multiple mechanisms are also believed to play a role in the anxiolytic-like activity of SGAs [16,17]. Finally, in the cerebral cortex and hippocampus of rodents, olanzapine and clozapine may increase the levels of GABAergic neuroactive steroid allopregnanolone [18], which is a potent GABA-A receptor modulator showing anxiolytic properties in several animal models, including the elevated plus-maze task [19,20].

Clinical studies on anxiety disorders have provided preliminary indications of anxiolytic effects of SGAs, especially quetiapine, in generalized anxiety disorder (GAD), even though issues with adverse effects (AEs) and tolerability suggested careful assessment of risks/benefits when considering the use of SGAs in these patients [21,22,23]. A recent review of the studies published until June 2013 suggested that SGAs (quetiapine, risperidone, olanzapine) could be effective and well-tolerated treatment options for PD, as either monotherapy or augmentation [24]. However, these results have primarily arisen from small, open-label studies, which do not allow drawing reliable conclusions. Therefore, we undertook an updated systematic review of all of the studies that have examined, in randomized controlled trials (RCTs), the efficacy and tolerability of SGAs in the treatment of PD, with or without other comorbid psychiatric disorders. We used the Preferred Reporting Items for Systematic Reviews and Meta-Analyses (PRISMA) guidelines [25].

## 2. Results

We included five studies in this review, which are summarized in Table 1. A recent proof-of-concept RCT [26] appraised the efficacy of quetiapine extended release (XR) (flexible doses) co-administration treatment compared to placebo (eight weeks) in a small sample of SSRI-/SNRI-resistant patients, with a principal diagnosis of PD and a Clinical Global Impression-Severity (CGI-S) scale score of ≥4. Most of them had comorbid psychiatric conditions. The method of defining SSRI/SNRI resistance was mixed, which is as follows: patients receiving adequate (≥8 weeks, in sufficient doses) ongoing SSRI/SNRI therapy at intake were classified as “resistant” if the psychiatrist’s clinical impression (Clinical Global Impression-Improvement (CGI-I)) was ≥3 (historical assessment); patients who were medication free at intake were treated for eight weeks with open-label sertraline (50–200 mg die), citalopram (20–40 mg die) or escitalopram (10–20 mg die); and patients who had a <50% decrease from baseline in the Panic Disorder Severity Scale (PDSS) total score after the eight-week SSRI treatment were classified as “resistant” (prospective assessment). No other psychotropic medications were allowed during the study, and surreptitious use of benzodiazepine was monitored by urine toxicology. During the trial, improvement of panic symptoms (primary outcome) and secondary outcome measures were observed in the whole sample, but no significant differences between quetiapine XR and placebo were found. Quetiapine XR was generally well tolerated, except for three patients who discontinued early due to medication-related SEs. No significant differences between quetiapine XR and placebo emerged in treatment-related SEs. According to the power calculation performed by the authors, this study was underpowered to detect small-to-moderate effects, while it was powered to detect large effect sizes.

Prosser and coworkers [27] compared the efficacy of monotherapy treatment with risperidone or paroxetine (eight weeks) in a mixed sample of patients with PD (43 patients, 76.8%) or with major depressive disorder (MDD) and PAs (13 patients, 23.2%). A baseline Hamilton Anxiety (HAMA) scale score of least 17 was required to be included, whereas no specific measures of panic symptomatology were used as inclusion/exclusion criteria. No other psychotropic medications were allowed during the study, even though no urine toxicology to monitor surreptitious use of drugs was reported. The two groups differed in the initiation of medications: risperidone was titrated, whereas paroxetine was not (paroxetine starting dose: 30 mg die). In the whole sample, the attrition rate of the participants was 48.2%. The rate of completers in the risperidone group was numerically higher than that in the paroxetine group, although the difference did not reach statistical significance. During the trial, a significant improvement of all of the outcome measures was found, including some panic symptom measures, without significant differences between risperidone and paroxetine. Of note, a significantly higher baseline severity of depressive symptoms in the paroxetine than in the risperidone group was found, with a significant correlation with some baseline and anxiety outcome measures, including midpoint PDSS total score. No separate analyses in the subgroup with PD were provided. No difference was found between the two groups in the number of participants who dropped out prematurely due to intolerable (unspecified) SEs, but, of note, the reason for attrition was not collected for seven participants who dropped out. Other information about SEs was not available. Finally, no power calculation was provided.

The following three studies focused on participants with bipolar disorder (BD) with co-occurring GAD or PD. An eight-week study [28] compared ziprasidone monotherapy with placebo in improving the clinical symptoms of patients with lifetime BD with co-occurring lifetime PD or GAD. At baseline, at least moderately severe anxiety symptoms (CGI-21 Anxiety Scale score ≥4) and not more than moderately severe bipolar symptoms (CGI-Bipolar Version <4) were required to be included in the study, whereas no specific measures of panic symptomatology were used as inclusion/exclusion criteria. The proportion of participants who had PD was not reported, and no analyses in the subgroup of participants with PD were provided. In the whole sample, the participant attrition rate was 53.1%. The rate of completers in the ziprasidone group was significantly lower than that in the placebo group. Some adjunctive medications were allowed during the study, but without a difference in distribution between the two groups. During the trial, a significant improvement in several outcome measures was found, including Sheehan Panic Scale (SPS) scores, but without significant differences between ziprasidone and placebo. According to the power calculation performed by the authors, this study was underpowered to detect small-to-moderate effects, while it had power to detect large effect sizes. Compared to placebo, a significantly higher number of participants in the ziprasidone group withdrew from the study due to AEs/SEs. Ziprasidone was associated with a significantly more negative SE profile than placebo.

An eight-week study [29] compared risperidone monotherapy with placebo in improving the clinical symptoms of patients with lifetime BD with co-occurring lifetime PD or GAD. At baseline, at least moderately severe anxiety symptoms (CGI-Severity Scale score ≥4) and not more than moderately severe bipolar symptoms (CGI-Bipolar Version ≤4) were required to be included in the study, whereas no specific measures of panic symptomatology were used as inclusion/exclusion criteria. Patients were excluded if they had psychotic symptoms. Both the proportion of participants who had lifetime PD (*n* = 80) and some analyses in the subgroup of participants with PD were provided, even though no analyses were performed on specific panic symptom scales in this subgroup. In the whole group, risperidone was not superior to placebo in reducing panic and anxiety symptoms, as well as in improving the other outcome measures. Within the subgroup with PD, placebo-treated patients had a significantly greater improvement on the global anxiety symptoms (HAMA scores) than those treated with risperidone. No power calculation was provided. Risperidone was well tolerated, with only one participant withdrawing due to an episode of heightened anxiety and anger. Extrapyramidal symptoms did not significantly differ between risperidone and placebo.

An eight-week study [30] compared quetiapine XR monotherapy with the mood stabilizer divalproex extended release (XR) and placebo in improving the clinical symptoms of patients with lifetime BD with co-occurring lifetime PD or GAD. At baseline, at least moderately severe anxiety symptoms (CGI-Severity Scale score ≥4) and not more than moderately severe bipolar symptoms (CGI-Bipolar Version ≤4) were required to be included in the study, whereas no specific measures of panic symptomatology were used as inclusion/exclusion criteria. Patients were excluded if they had current psychotic symptoms or lifetime psychotic disorders. Both the proportion of participants who had current PD (*n* = 113) and some analyses in the subgroup of participants with PD were provided, including a panic symptom measure (SPS scores). In the whole group, the mean baseline-to-endpoint improvement was significantly greater for quetiapine XR compared to both divalproex XR and placebo on the HAMA (Hamilton Anxiety Scale) and SPS (Sheehan Panic Scale) scores, while only a trend of significance was found on the CGI-21 Anxiety Scale score. The effects of baseline depression severity on the HAMA and SPS outcome scores was not evaluated, even though, of note, at the study endpoint, the improvement on CGI-21 Anxiety Scale score in the quetiapine XR group was significantly higher in patients with lower baseline depression severity than in those with higher baseline depression severity. In the subgroup with current PD, a global model of repeated-measure ANOVA showed a significant improvement in both HAMA and SPS scores at the end of the study. However, after *post hoc* comparisons between the treatment groups, the authors reported a sole significant result, *i.e.*, that quetiapine XR was significantly (*p* < 0.05) superior to divalproex extended release XR in improving HAMA and SPS scores, whereas they did not report any significant differences between quetiapine and placebo. Additional analyses in the whole group showed significantly higher response rates (defined as ≥50% improvement on CGI-21 Anxiety Scale score or alternately as a ≥50% reduction on HAMA score at study endpoint) for those treated with quetiapine XR compared to divalproex XR (*p* < 0.01), but not with placebo. Remission rate (defined as ≥70% improvement on CGI-21 Anxiety Scale score at study endpoint) was significantly higher for those treated with quetiapine XR compared to divalproex XR (*p* < 0.02), but not with placebo, whereas remission rate defined as a ≥70% reduction on HAMA score at study endpoint did not differ significantly between the groups. Both active medications were well tolerated. Only one participant in the quetiapine XR group and three in the divalproex XR group withdrew due to medication-related AEs. Participants treated with quetiapine XR reported significantly higher rates of dry mouth compared to both divalproex XR and placebo, while those treated with quetiapine XR and divalproex XR reported greater weight gain compared to placebo. Extrapyramidal symptoms did not differ significantly by the treatment group.

### Risk of Bias in Individual Studies

Table 2 provides a summary of the possible risks of bias across all of the reviewed studies. In terms of selection bias, only one study presented a low risk of bias in random sequence generation, while in the other four studies, insufficient information about a random component in the sequence generation did not permit a judgment (unclear risk). A low risk of allocation concealment was found in two studies, while in the other three studies, the risk was unclear. In terms of performance and detection bias, two studies were at low risk in the blinding of participants, one was at high risk (single-blind design: participants not blinded), while in the other two studies, the risk was unclear; only one study was at low risk in both blinding of personnel and blinding of outcome assessors, while in all of the other studies, the risk was unclear in both of these domains. In terms of attrition bias, two studies were at low risk and three were at high risk. In terms of reporting bias, all of the studies were at low risk. In terms of sampling bias, two studies were at high risk in recruitment strategies; one was at low risk; and in the other two studies, the risk was unclear; while all of the studies were at high risk in the inclusion criteria. In terms of other biases, two studies were at high risk in detecting small-to-moderate effect sizes, while in the other three studies, the risk in the power calculation domain was unclear; four studies were at low risk for bias in the domain of adjunctive medications, while in the other one, the risk was unclear. Finally, two studies were at high risk for some adjunctive biases.

## 3. Discussion

Based on the PRISMA guidelines [25], we provided a systematic review of the RCTs investigating the efficacy and tolerability of SGAs (as either monotherapy or augmentation) in the treatment of PD, with or without other comorbid psychiatric disorders. A very limited number of studies were available; all were short term-trials (eight weeks) and involved treatments with quetiapine XR, risperidone and ziprasidone. Among the five studies included, two involved patients with a principal diagnosis of PD, while the other three studies involved patients with BD with co-occurring PD or GAD. Overall, a general lack of efficacy of SGAs on panic symptoms was observed. Some preliminary indications of the antipanic effectiveness of risperidone are insufficient to support its use in PD, primarily due to major limitations of the study. However, several methodological limitations, with the related risks of bias, may have negatively affected all of these studies, decreasing the validity of the results and making it difficult to draw reliable conclusions. In the short time frame of these RCTs, SGAs were well tolerated, except for ziprasidone.

### 3.1. Efficacy in Patients with a Principal Diagnosis of PD

The quetiapine XR augmentation strategy was not different from placebo in improving either specific panic symptoms or general anxiety symptoms in a sample of SSRI-/SNRI-resistant patients with PD and with other comorbid psychiatric conditions [26]. The sample size was very small and powered to detect large effect sizes, while it was underpowered to detect small-to-moderate effects. The primary limitation of this study was the mixed and unstandardized method of defining SSRI/SNRI resistance. This led to a highly heterogeneous sample that included patients taking multiple medications at different doses and for different time frames, some of whom have been selected with prospective assessment and psychometric tools specific for panic symptoms, while others with historical assessment and psychiatrist’s clinical impression of improvement. This may have exerted confounding effects on the results. For instance, in some patients, SSRI/SNRI treatment could not have still expressed its antipanic effects at the beginning of the study, while it may have expressed them during the augmentation trial, masking the possible differences between quetiapine and placebo. Furthermore, the high variety of psychiatric comorbidities may have influenced the treatment outcome. Thus, these negative results about large treatment effects of quetiapine XR in SSRI-/SNRI-resistant patients with PD need to be confirmed by employing a more rigorous definition for treatment resistance and inclusion/exclusion criteria. It should be noted that, despite a considerable rate of patients with PD who do not achieve full remission with the recommended drugs and some open studies that suggested the efficacy of SGAs as monotherapy or augmentation strategy in treatment-resistant PD [5,24], we found only one RCT on this topic. This may be partly related to the lack of consensus about criteria for defining treatment resistance in PD [5] and to the existence of several pharmacological/nonpharmacological options for this disorder.

Low-dose risperidone as monotherapy showed similar efficacy to that of paroxetine in improving both panic symptoms and general anxiety/depressive symptoms in a mixed sample of patients with PD (without other psychiatric comorbidities) and patients with MDD and PAs [27]. However, the reliability of these positive effects of risperidone was negatively affected by several risks of bias. MDD diagnosis in approximately 23% of the patients may have influenced the results, and no separate analyses in the subgroup with PD were available. The unbalanced severity of baseline depressive symptoms (higher in the paroxetine group) and its positive correlation with baseline/outcome measures of general anxiety and panic symptoms do not allow an understanding of whether the anxiolytic/antipanic effects of risperidone were secondary to the decrease of depression severity. No gradual titration has been used for paroxetine, contrary to the current guidelines that recommend very low starting doses in patients with PD, who are particularly sensitive to SEs and SSRI-induced anxiety/panic symptoms at the beginning of treatment [31]. This may have contributed to the very low rate of completers in the paroxetine group and may have elicited a paroxetine-induced increase of symptoms, thus affecting the outcome measures and masking the possible differences between the two treatment groups. In addition, no information about withdrawal from previous medications was reported. The attrition rate was high (about half of the sample), and no power calculation was provided, thus decreasing the validity of the results. Finally, the heterogeneity of the sample may have influenced the results. Indeed, it cannot be excluded that Pas in patients with MDD and full-blown PD may be related to different mechanisms, with a possible different response to treatments. Our team found that subjects with Pas, who did not meet the criteria for PD, showed a behavioral hypersensitivity to 35% CO_2_ inhalation, similar to patients with PD [32]; however, on the other hand, the development of sporadic Pas did not seem to share a common genetic vulnerability with PD [33], thus making unclear if sporadic Pas and PD belong to the same spectrum of vulnerability.

In conclusion, an augmentation treatment with quetiapine XR did not show efficacy in SSRI-/SNRI-resistant patients with a principal diagnosis of PD, although confirmations are required, while there is insufficient evidence to recommend the use of risperidone as monotherapy in PD.

### 3.2. Efficacy in Patients with BD with Co-Occurring PD

Compared to placebo, ziprasidone as monotherapy was not associated with significant improvement of either general anxiety or panic symptoms in a mixed sample of patients with BD, with co-occurring PD or GAD, powered to detect large effect sizes [28]. Several limitations negatively affect the reliability of these results, primarily the high attrition rate (almost half of the sample), the significantly higher proportion of ziprasidone-treated patients who withdrew from the study due to AEs/SEs and the heterogeneity of the sample. Since there is evidence of qualitative differences and diverse pathogenetic mechanisms between PD and GAD [34], these two disorders may have different pharmacological responses. A heterogeneous sample, without separate analyses in the subgroup with comorbid PD, did not allow appraising specific antipanic properties of ziprasidone. However, the significantly more negative SE profile of ziprasidone, compared to that of placebo, discourages subsequent studies to test its antipanic efficacy. Likewise, in a similar sample, risperidone as monotherapy, compared to placebo, was not associated with significant improvement of either general anxiety or panic symptoms [29], while in the subgroup with comorbid PD, placebo showed better anxiolytic properties than risperidone. The lack of power calculation, the high attrition rate, the unbalanced baseline features of the two groups (higher proportion of participants with mixed mood state and with PD in the risperidone group) and the lack of analyses on specific panic symptom measures in the subgroup with comorbid PD make it difficult to draw reliable conclusions about the antipanic properties of risperidone in this population.

In a similar sample [30], quetiapine XR as monotherapy showed, in the subgroup with comorbid PD, significantly higher efficacy in improving both general anxiety and panic symptoms when compared to the mood stabilizer divalproex XR. However, because the authors did not report any significant comparison between quetiapine XR and placebo, we interpreted this as a lack of differences between quetiapine XR and placebo in improving symptoms in this subgroup of patients. Since in the whole group, higher baseline depression severity was associated with higher general anxiety severity at the endpoint, a possible influence of baseline depression severity on the outcomes in the comorbid PD subgroup cannot be excluded and should have been investigated. Finally, a power calculation should have been provided.

In conclusion, neither ziprasidone, risperidone nor quetiapine XR as monotherapy seemed to show higher efficacy than placebo in improving panic symptoms in patients with BD with comorbid PD, while quetiapine XR displayed higher efficacy than divalproex XR in improving both panic and general anxiety symptoms in this population. However, several methodological deficiencies of these RCTs have compromised the reliability of their results.

### 3.3. Tolerability

Risperidone and quetiapine XR were generally well tolerated in these short time frame RCTs, whereas ziprasidone showed an unfavorable SE profile. In only one study, quetiapine XR induced greater weight gain than placebo, even after eight weeks [30]. However, in a longer time frame, the risk of metabolic dysregulation, including insulin resistance, dyslipidemia and hyperglycemia, as well as weight gain and tardive dyskinesia has been reported, suggesting caution in their use and a close monitoring of SEs [35,36]. In PD, recent indications have suggested to continue the recommended pharmacotherapy for at least 6–12 months after the acute response and even longer than 12 months if the disorder is recurrent or particularly severe [21,31,37,38]. The need for long-term treatment in PD casts doubt on the risk-to-benefit ratio of using SGAs in PD, when compared to the existing treatments for this disorder, especially in patients with a principal or sole diagnosis of PD.

### 3.4. Limitations and Future Research

Our review of the few RCTs investigating the antipanic effects of SGAs did not provide reliable support for the use of quetiapine XR, risperidone or ziprasidone in patients with a principal diagnosis of PD or in patients with BD with comorbid PD, although the global methodological weakness of the studies limits the inferences and interpretations that can be made from these results. These conclusions cannot rule out that better designed RCTs may find more favorable results or that other SGAs, with different pharmacological profiles and receptor selectivity, may have an antipanic efficacy. The antipanic properties of SGAs may be especially worthy of being investigated in some challenging clinical conditions, such as in patients with PD resistant to multiple recommended treatments or, primarily, in patients with BD with comorbid PD, whose treatment represents a therapeutic challenge. PD is highly prevalent in patients with BD (the lifetime rate is about 30%) [39], and there is evidence that BD with comorbid PD may represents a distinct phenotype, with unique genetic vulnerability [40,41]. Comorbid panic is associated with higher severity of BD, including increased risk of suicide [42], higher frequency/severity of depressive episodes [43], longer time to remission [44], poorer responses to antidepressants [45] and more severe medication-related SEs [44]. Since the antidepressant drugs recommended for panic may induce mania and rapid cycling in patients with BD [46], it could be meaningful to further investigate the potential antipanic effectiveness of SGAs in this peculiar population with BD and comorbid PD, considering that SGAs are already used and recommended in BD [47].

To date, no definite conclusions can be drawn; future research should consider and overcome the methodological weakness of the available studies. Beyond the limitations discussed above, other relevant issues need consideration. None among the reviewed RCTs used panic symptom severity as the inclusion criterion, whereas only measures of general anxiety or clinical global impression were employed, with a high risk of sampling bias. Anxiety symptoms are also present in PD, but they are considered qualitatively distinct from PAs and related to different biological mechanisms [13,14,48,49]. Thus, to draw conclusions about the antipanic efficacy of SGAs, it is mandatory that future studies also incorporate specific pretreatment panic measures. Similarly, outcome measures should include psychometric tools that could assess separately unexpected/expected PAs, anticipatory anxiety and phobic avoidance. This allows disentangling potential medication effects on different clinical phenomena of PD, which may be masked by the use of total panic-phobic symptom scores. For instance, since preclinical studies showed effects of SGAs on fear conditioning processes [8,9,10], these compounds may exert some therapeutic effects on the conditioned anxiety and phobic behaviors of patients with PD [11,12,13]. To date, no preclinical or clinical studies tested the ability of SGAs to block CO_2_- or sodium lactate-induced PAs, the core symptom of PD, although panic can be reliably provoked in the laboratory by validated methods (e.g., CO_2_ inhalation or sodium lactate infusion). Since hypersensitivity to CO_2_/sodium lactate is considered a biomarker of vulnerability to PAs, incorporating these procedures in preclinical/clinical studies may help to understand if SGAs may be really effective on PAs. To date, a biomarker/endophenotype-based approach has been scantily employed in pharmacological studies on PD, although it may help to reduce the variability within the nosographic diagnosis and the heterogeneity, both in samples and in results. Hypersensitivity to hypercapnia is considered an endophenotype of panic and is associated with respiratory symptoms, higher frequency of PAs and familiarity for PD and is related to genetic factors, probably characterizing a respiratory-panic subtype. Patients with PD have shown several abnormalities in their respiratory/autonomic/balance system functions, which may lead to different clinical symptoms and outcomes [3,7]. Endophenotypes or patterns of neurobiological functions/clinical features in pharmacological studies may offer advantages in selecting truly homogeneous patients, identifying more appropriate targets and outcomes and testing the effectiveness of compounds on specific symptoms and functions. In the age of personalized medicine aimed to tailor medications in order to maximize therapeutic effectiveness and minimize SEs according to each patient’s unique characteristics, it is necessary to re-think RCTs. They usually concern the “average” patient, who often does not correspond to real-life patients with their peculiar features. Future pharmacological studies should identify evidence-based predictors of treatment response and tolerability, to select those patients who may mostly benefit from a specific treatment. Personalized treatments may be carried out with predictive tools [50] to identify those variables influencing the heterogeneity of treatment response/tolerability, such as gender, familiarity, clinical features, comorbidity, neurobiological functions, biomarkers and genetic/pharmacogenetic characteristics, and to select for each patient the most proper medications for effectiveness, tolerability and length of treatment [51,52]. The less use of this approach may also partly explain why some patients with PD do not seem to respond adequately to recommended pharmacotherapy. Only limited investigations have found that allelic variations of serotonergic system genes may influence the short-term outcomes of SSRI treatment in PD [52], and the decrease of hypersensitivity to hypercapnia after the first week of treatment with TCAs or SSRIs was a significant predictor of good clinical outcome after one month [53]. The development and the wide application of predictive tools could increase the rate of responders to the available recommended treatments for PD. Furthermore, this approach is mandatory for non-recommended treatments with a potentially unfavorable SE profile, such as SGAs, whose use needs a careful assessment of risks/benefits. In the realm of PD, for which several pharmacological/nonpharmacological options exist and whose outcome may be improved by the personalized medicine strategy, future studies on SGAs should demonstrate reliably whether and for what peculiar patient with PD their use has a favorable cost-benefit ratio. Our review has shown that, to date, no sufficient evidence supports their usefulness in this disorder.

## 4. Materials and Methods

**Figure 1:**
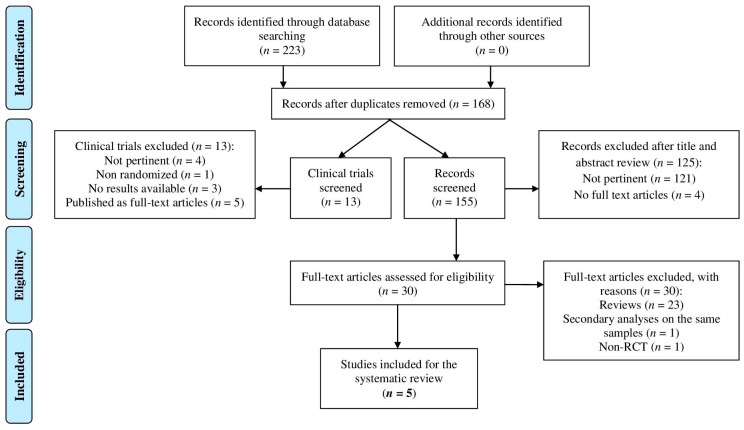
PRISMA flow diagram of study selection process.

This systematic review was performed according to the PRISMA guidelines [25]. The protocol for this review has not been previously registered, and the search strategy has not underwent any peer reviews. A database search of scientific literature, written in English, on RCTs until 31 December 2015 was performed through PubMed, PsycINFO, Embase, Cochrane Library and Clinical Trials.gov. The following search terms were used: (“panic” AND ((atypical antipsychotic *) OR (second generation antipsychotic *) OR (dopamine receptor antagonist *) OR (neuroleptic *) OR (major tranquilizer *) OR (serotonin-dopamine antagonist *) OR “quetiapine” OR “ziprasidone” OR “risperidone” OR “olanzapine” OR “clozapine” OR “aripiprazole” OR “amisulpride” OR “asenapine” OR “paliperidone” OR “sulpiride” OR “sertindole” OR “zotepine” OR “iloperidone” OR “lurasidone” OR “melperone” OR “blonanserin”)) AND (random * OR RCT). We used asterisks (*) in order to search for multiple characters after a search string. We also used the reference lists of relevant studies and pertinent review articles to gain access to additional literature. Among 223 records identified in the search, five studies were included in the review (Figure 1, PRISMA flow diagram). The population, interventions, comparators, outcomes and study design approach (PICOS) [25] was followed to determine the eligibility criteria of the studies for this systematic review. Studies were included in the review if they had included participants who were ≥18 years of age, with a diagnosis of PD identified through a structured, clinician-administered interview conducted according to the Diagnostic and Statistical Manual of Mental Disorders criteria, Third Edition (DSM-III/DSM-III-R) [54,55], Fourth Edition (DSM-IV/DSM-IV-TR) [56,57], or to the International Classification of Diseases criteria, Ninth Edition (ICD-9/ICD-9-CM) [58,59] and Tenth Edition (ICD-10) [60], or through clinical unstructured interviews, but only if the diagnosis was in accordance with the diagnostic criteria listed above; pharmacological interventions with SGAs, as either monotherapy or augmentation of existing pharmacological treatments; comparators including placebo and/or active comparators; validated self-reported and/or clinician-administered psychometric scales as efficacy outcome measures; safety assessment; if they had a randomized (double- or single-blind) controlled design; if full texts were available; and if, for studies on Clinical Trials.gov, the RCTs have not been published yet as full articles and all of the details were available to satisfy the PICOS’ items.

The PRISMA flow diagram (Figure 1) provides detailed information regarding the selection process of the studies. Each step of the search and selection procedures was performed independently by two authors, and inconsistency in the results was discussed and resolved before proceeding.

An assessment of the risk of bias across all of the reviewed studies was conducted using the Cochrane Collaboration tool [25,61]. The following domains have been considered: (i) selection bias, including biases due to inadequate random sequence generation and inadequate allocation concealment; (ii) performance bias, including biases in the blinding of participants and blinding of personnel; (iii) detection bias, referring to bias in the blinding of outcome assessors; (iv) attrition bias, referring to biases due to the amount/nature/handling of incomplete outcome data; and (v) reporting bias, due to selective outcome reporting and/or not reporting relevant outcomes that would have been expected to be reported. In addition, other sources of bias relevant to the aim of this review were considered: (i) sampling bias, including biases resulting from recruitment strategies and inclusion/exclusion criteria used; and (ii) other bias, including biases resulting from power calculation and adjunctive medications or adjunctive bias, *i.e.*, other particular biases. Review authors’ judgments were categorized as “low risk” of bias, “high risk” of bias or “unclear risk” of bias (*i.e.*, when insufficient information did not permit a judgment of “low risk” or “high risk”). The assessment of risk of bias was performed independently by two authors, and inconsistency in the results was discussed and resolved.

## 5. Conclusions

Our systematic review of the few RCTs investigating the antipanic effects of SGAs has shown that, till date, no sufficient evidence supports the use of quetiapine XR, risperidone, or ziprasidone either in patients with a principal diagnosis of PD or in patients with BD with comorbid PD. Risperidone and quetiapine XR were generally well tolerated in these short time frame RCTs, whereas ziprasidone showed an unfavorable side effect profile. However, the global methodological weakness of the studies limits the inferences and interpretations that can be made from these results.

## Figures and Tables

**Table 1:** Randomized controlled trials with second-generation antipsychotics in panic disorder.

**Authors, Year [Reference]**	**Study Design**	**Duration**	**Recruitment**	**Participants**	**Other Psychiatric Diagnoses (Number of Participants)**	**Psychiatric Assessment Instruments**
Goddard *et al.*, 2015 [26]	Single-site, double-blind, placebo-controlled, randomized, quetiapine XR (flexible-dose) coadministration trial	8 weeks	Mixed strategies (referrals from local clinicians, flyers in the university hospital, on-line bulletins, ads in local newspapers)	Patients with primary, current PD with or without AG, SSRI-/SNRI-resistant	In the ITT patients: GAD (*n* = 8), PTSD (*n* = 3), MDD (*n* = 8), depression NOS (*n* = 2), dysthymia (*n* = 1), ADD (*n* = 1), bulimia (*n* = 1)	Mini-International Neuropsychiatric Interview (MINI Plus) for DSM-IV. Qualification of interviewer: not reported
Prosser *et al.*, 2009 [27]	Single-site, single (*i.e.*, rater)-blind, medication-controlled, flexible doses, randomized	8 weeks	Mixed strategies (recruitment from inpatients psychiatric units, a psychiatric outpatient service, ads in local newspapers and an Internet website)	Patients with PD, with or without AG; patients with MDD and PAs	None; patients with any other current/lifetime Axis I diagnosis were excluded	Unstructured psychiatric clinical interview conducted by experienced psychiatrists, according to DSM-IV criteria
Suppes *et al.*, 2014 [28]	Three-site, double-blind, placebo-controlled, flexible doses, randomized	8 weeks	Outpatients	Patients with lifetime BD (I/II/NOS) and with lifetime PD, with or without AG or GAD	Not reported	Structured Clinical Interview for DSM-IV-TR (SCID). Qualification of interviewer: not reported
Sheehan *et al.*, 2009 [29]	Three-site, double-blind, placebo-controlled, flexible doses, randomized	8 weeks	Outpatients recruited from three University sites by ads	Patients with lifetime BD (I/II/NOS) and with lifetime PD, with or without AG or GAD	Not reported	Mini-International Neuropsychiatric Interview (MINI Plus) for DSM-IV. Qualification of interviewer: not reported
Sheehan *et al.*, 2013 [30]	Three-site, double-blind, placebo-controlled, flexible doses, randomized	8 weeks	Outpatients	Patients with lifetime BD (I/II/not otherwise specified) and with lifetime PD, with or without AG or GAD	Not reported	Mini-International Neuropsychiatric Interview (MINI Plus) for DSM-IV. Qualification of interviewer: not reported
**Authors, Year [Reference]**	**Number of Randomized Patients**	**Treatments (*n* = Number of Participants)**		**Daily Dose Range (mg)**	**Mean Daily Dose (mg, (SD) or Range)**	**Completer Population (*n* = Number of Participants)**
Goddard *et al.*, 2015 [26]	27	Adjunctive treatment to SSRI/SNRI stable dose (*i.e.*, baseline SSRI/SNRI doses were held constant throughout the 8-week trial): quetiapine XR (*n* = 14), PLB (*n* = 13). No other psychotropic medications were allowed during the study (urine toxicology: yes)		Quetiapine XR 50–400	150 (106)	*n* = 21 (78% of the randomized group)
Prosser *et al.*, 2009 [27]	56	Monotherapy: risperidone (*n* = 33) or paroxetine (standard-of-care) (*n* = 23). The observed randomization distribution did not significantly deviate from the expected distribution on a 1: 1 basis (*i.e.*, 28 subjects per treatment group). No other psychotropic medications were allowed during the study (urine toxicology: no). Period of withdrawal from previous medication: not reported		Risperidone: 0.125–16, paroxetine: 30–60	Risperidone: 0.53 (range 0.125–1.0), paroxetine: all participants received 30 mg, except one who received 40 mg	*n* = 29 (51.8% of the randomized group). Risperidone: *n* = 20 (60.6%); paroxetine: *n* = 9 (39.1%) (no significant difference in the retention rate between the two treatment groups)
Suppes *et al.*, 2014 [28]	49	Monotherapy: ziprasidone (*n* = 25, % female = 76, mean age (SD) = 36.7 (17.7) years), PLB (*n* = 24, % female = 70.8, mean age (SD) = 34.6 (12.2) years). Participants discontinued any psychotropic medication for at least 1 week before baseline (if fluoxetine or depot antipsychotics: for at least 4 weeks). Adjunctive lorazepam (up to 2 mg die) allowed during the first 2 weeks of the study. Zolpidem/zaleplon for insomnia and benztropine for EPs allowed throughout the study		Ziprasidone 40–160	146.7 (20.7)	*n* = 23 (46.9% of the randomized group). Ziprasidone: *n* = 6 (24%); PLB: *n* = 17 (70.8%) (*p* = 0.001)
Sheehan *et al.*, 2009 [29]	111	Monotherapy: risperidone (*n* = 54, % female = 66.7, mean age (SD) = 35.1 (12.4) years, participants with lifetime PD: *n* = 45, 83.3%), PLB (*n* = 57, % female = 61.4; mean age (SD) = 38.4 (12.8) years, participants with lifetime PD: *n* = 35, 61.4%). Participants discontinued any psychotropic medication for at least 1 week before baseline (if fluoxetine or depot antipsychotics: for at least 4 weeks). Adjunctive lorazepam allowed during the first 1 week (up to 2 mg die) and the second week (up to 1 mg die) of the study. Zolpidem/zaleplon for insomnia allowed throughout the study		Risperidone 0.5–4	2.5 (1.1)	*n* = 63 (56.7% of the randomized group). Risperidone: *n* = 27 (50%); PLB: *n* = 36 (63%)
Sheehan *et al.*, 2013 [30]	149	Monotherapy: quetiapine XR (*n* = 49, % female = 57.1, mean age (SD) = 41.4 (12.1) years, participants with current PD: *n* = 37), divalproex XR (*n* = 49, % female = 55.1%, mean age (SD) = 37.5 (12.0) years, participants with current PD: *n* = 37), PLB (*n* = 51, % female = 64.7; mean age (SD)= 37.6 (11.6) years, participants with current PD: *n* = 39). Participants discontinued any psychotropic medication for at least 1 week before baseline (if fluoxetine or depot antipsychotics: for at least 4 weeks). Adjunctive lorazepam allowed during the first 1 week (up to 2 mg die) and the second week (up to 1 mg die) of the study. Zolpidem/zaleplon for insomnia allowed throughout the study		Quetiapine XR 50–300, divalproex XR 500–3000	Quetiapine XR: 186.4 (100.3), divalproex XR: 1991 (866)	*n* = 108 (72.5% of the randomized group). Quetiapine XR: *n* = 38 (77.5%); divalproex XR: *n* = 35 (71.4%); PLB: *n* = 35 (68.6%)
**Authors, Year [Reference]**	**ITT Population (*n* = Number of Participants)**		**Significant Baseline Differences in Socio-Demographic/Clinical Characteristic between Treatment Groups**		**Main Outcome Measures and Results**	
Goddard *et al.*, 2015 [26]	*n* = 26 (quetiapine XR, *n* = 13, % female = 77; mean age (SD) = 35.5 (9.6) years; PLB, *n* = 13, % female = 62, mean age (SD) = 35.5 (16.8) years). LOCF imputation was used for participants who withdrew prematurely		None		PDSS total scores; PDSS Item 1 (panic attack frequency) score; rates of responders (*i.e.*, ≥50% improvement from baseline PDSS total score); rate of remitters (*i.e.*, PDSS total score ≤4) at endpoint. Both in the ITT and completer populations: significant improvement in panic symptoms over the trial (main effect of time, *p* < 0.0001), but no significant drug/PLB differences.	
Prosser *et al.*, 2009 [27]	*n* = 56 (risperidone, *n* = 33, % female = 76; mean age (SD) = 38.8 (9.7) years, PD diagnosis *n* = 24 (73%); paroxetine, *n* = 23, % female = 65, mean age (SD) = 42.6 (14.3) years, PD diagnosis *n* = 19 (83%). LOCF imputation was used for participants who withdrew prematurely		Significantly higher HAM-D scores in the paroxetine group than risperidone group (*p* = 0.049)		CGI, HAMA, HAMD scores, PDSS total scores, PDSS Items 1 (panic attack frequency) and 2 (panic attack severity) scores, SPAS-P score. In the ITT population: significant improvement in all of the outcome measures over the trial, except for SPAS-P score. No significant differences between risperidone and paroxetine. Baseline HAMD scores significantly correlated with both baseline and midpoint/endpoint outcome HAMA scores and with midpoint outcome PDSS total scores. No analyses in completer population. No analyses in the subgroup with PD.	
Suppes *et al.*, 2014 [28]	*n* = 46 (ziprasidone, *n* = 23; PLB, *n* = 23). LOCF imputation was used for participants who withdrew prematurely		Significantly higher SSTS scores in the ziprasidone group than PLB group (*p* = 0.037)		CGI-21 Anxiety, SDS scores. In the ITT population: significant improvement in both measures over the trial, but no significant differences between ziprasidone and PLB. No analyses in completer population. No analyses in the subgroup with PD.	
Sheehan *et al.*, 2009 [29]	*n* = 102 (risperidone, *n* = 49; PLB, *n* = 53). LOCF imputation was used for participants who withdrew prematurely		Significantly higher rate of participants with a mixed mood state (59% *vs*. 40%, *p* < 0.05) and lifetime PD (83.3% *vs*. 61.4%, *p* < 0.01) in the risperidone group than the PLB group		CGI-21 Anxiety score. No difference in improvement between risperidone and PLB. Over the trial, the improvement was similar for patients with and without PD. Within the subgroup with PD, the PLB group showed a trend towards greater improvement (*p* < 0.07). No analyses in the completer population.	
Sheehan *et al.*, 2013 [30]	*n* = 144 (quetiapine XR *n* = 47, divalproex XR *n* = 46; PLB, *n* = 51). LOCF imputation was used for participants who withdrew prematurely		None		CGI-21 Anxiety score. The quetiapine XR group had a numerically higher improvement compared to divalproex XR and PLB, but it did not reach statistical significance (*p* < 0.07). No analyses in the completer population were available.	
**Authors, Year [Reference]**	**Secondary Outcome Measures and Other Results**		**Side Effects/Tolerability**			**Funding**
Goddard *et al.*, 2015 [26]	CGI-S, CGI-I, HAMA, HAMD, PSQI scores: significant improvement over the trial (*p* < 0.0001; PSQI (sleep quality item) *p* < 0.05), but no significant drug/PLB differences.		Three patients in the quetiapine XR group discontinued early due to medication-related SEs (sedation, *n* = 1; derealization, *n* = 2). No significant difference in SEs emerged between quetiapine XR and PLB, including sedation/somnolence, extrapyramidal SEs and akathisia (BARS, SAS), weight gain and blood glucose levels.			AstraZeneca
Prosser *et al.*, 2009 [27]	Preliminary evidence of significantly faster decrease of HAMA and HAMD in the risperidone group than the paroxetine group (*t*-tests, without corrections for multiple comparisons).		No significant difference between the two groups in the number of participants who discontinued early due to intolerable (unspecified) SEs (*n* = 2 in the risperidone group; *n* = 1 in the paroxetine group). The reason for attrition was not collected for 7 participants who discontinued early. No other information available on SEs.			Partial support by a grant from New York State Empire Clinical Research Investigation Award
Suppes *et al.*, 2014 [28]	PGI-21, HAMA, SPS, CGI-BP, YMRS, MADRS, SSTS, SIS scores. No significant differences between ziprasidone and PLB.		Compared to PLB, a significantly higher number of participants in the ziprasidone group withdrew from the study due to AEs/SEs (*n* = 9 and 2, respectively, *p* = 0.02). The ziprasidone group reported significantly more sleep disturbance (*p* = 0.040), sedation/somnolence (*p* = 0.049), weight gain (*p* = 0.035) and higher increase of the AIMS scores (*p* = 0.003) than PLB. No difference in akathisia (BARS) was found between the two groups.			Pfizer
Sheehan *et al.*, 2009 [29]	SPS, HAMA, PG-21 Anxiety, YMRS, IDS, CGIBP, SDS scores. No difference between risperidone and PLB in improvement on all outcome measures. Over the trial, improvement of the HAMA score was similar for patients with and without PD. Within the subgroup with PD, the PLB group had a significantly lower mean endpoint HAMA score than the risperidone group (*p* < 0.007), but no analyses on the specific panic symptom scale (SPS) were available.		One participant in the risperidone group and one in the PLB group discontinued early due to treatment-related adverse events (risperidone: one episode of heightened anxiety and anger; PLB: multiple symptoms). Drowsiness was the only side effect that was two times more frequent in the risperidone group than the PLB group. Extrapyramidal symptoms (BARS, SAS, AIMS) did not significantly differ between the two groups. Weight gain was numerically higher in the risperidone group, but without statistical significance.			Janssen Pharmaceutica
Sheehan *et al.*, 2013 [30]	HAMA, SPS, PGI-21 Anxiety, YMRS, MADRS, CGIBP, SIS, RISC, SSTS, SDS scores. The quetiapine XR group had a significantly greater improvement on HAMA and SPS compared to both divalproex XR and PLB groups (*p* < 0.05). No significant differences between groups were found on PGI-21 Anxiety, YMRS, SSTS, SIS and RISC scores. The quetiapine XR group had a significantly greater improvement on the MADRS score compared to both divalproex and PLB groups (*p* < 0.05) and on the SDS and CGIBP depression scores compared to divalproex XR (*p* < 0.05 and *p* < 0.04, respectively). In the subgroup with current PD (*n* = 113), the quetiapine XR group had a significantly greater improvement on HAMA and SPS compared to the divalproex XR group (*p* < 0.05).		One participant in the quetiapine XR group, 3 in the divalproex XR group and one in the PLB group discontinued early due to treatment-related adverse events. The most common SEs in the three groups were: drowsiness, dry mouth, nausea, tingling, increased appetite, sedation, headache. Participants in quetiapine XR reported significantly higher rates of dry mouth compared to both divalproex XR and PLB (*p* < 0.006). Participants in quetiapine XR and divalproex XR reported greater weight gain compared to PLB (*p* < 0.001, *p* < 0.03, respectively). Extrapyramidal symptoms (BARS, SAS, AIMS) did not significantly differ by treatment group.			AstraZeneca

ADD = attention deficit disorder; AE(s) = adverse event(s); AG = agoraphobia; AIMS = abnormal involuntary movement scale; AnxDs = anxiety disorders; BARS = Barnes Akathisia Rating Scale; BD = bipolar disorder; CGI = Clinical Global Impression Scale; CGI-21 = Clinical Global Impression-21 Items; CGI-BP = Clinical Global Impression-Bipolar; CGI-I = Clinical Global Impression-Improvement Scale; CGI-S = Clinical Global Impression-Severity Scale; EPs = extrapyramidal symptoms; GAD = generalized anxiety disorder; HAMA = Hamilton Anxiety Rating Scale; HAMD = Hamilton Depression Rating Scale; IDS = Inventory of Depressive Symptoms; ITT = intention-to-treat (defined as all participants who received at least 1 dose of study medication and had at least 1 post-baseline assessment); LOCF = last observation carried forward; MADRS = Montgomery–Asberg Depression Rating Scale; MDD = major depressive disorder; NOS = not otherwise specified; PAs = panic attacks; PD = panic disorder; PDSS = panic disorder severity scale; PGI-21 = Patient Global Impression of Change-21 Items; PSQI = Pittsburgh Sleep Quality Index; PLB = placebo; PTSD = post-traumatic stress disorder; RISC = rapid ideas scale; SAS = Simpson–Angus Scale; SD = standard deviation; SDS = Sheehan Disability Scale; SEs = side effects; SIS = Sheehan Irritability Score; SNRIs = serotonin norepinephrine reuptake inhibitors; SPAS-P = Sheehan Panic Anxiety Scale-Patient (self-reported); SPS = Sheehan Panic Scale; SSRIs = selective serotonin reuptake inhibitors; SSTS = Sheehan Suicide Tracking Score; XR = extended release; YMRS = young mania rating scale.

**Table 2:** Risk of bias in individual studies.

**Authors, Year**	**Selection Bias**		**Performance Bias**		**Detection Bias**	**Attrition Bias**	**Reporting Bias**
	**Random Sequence Generation**	**Allocation Concealment**	**Blinding of Participants**	**Blinding of Personnel**	**Blinding of Outcome Assessors**	**Incomplete Outcome Data**	**Selective Reporting**
Goddard *et al.*, 2015 [26]	U Participants were randomized sequentially.	L Pharmacy-controlled randomization; identical-appearing PLB/quetiapine tablets; coordinator (not involved in patients ratings) managed the medication bottles.	L	L	L	L	L
Prosser *et al.*, 2009 [27]	L Computer number random generator (SPSS 12.0.1).	U	H Participants not blinded.	U	U	H High attrition rate.	L
Suppes *et al.*, 2014 [28]	U Randomized block design.	U	U Blinded guess of treatment performed. Participants were accurate 71.4% of the time; those in the PLB group and the ziprasidone group were accurate 100% and 66.7% of the time, respectively.	U Blinded guess of treatment performed. Treating clinicians were accurate 47.6% of the time; within the ziprasidone group: 66.7% of the time.	U Blinded guess of treatment performed. Raters were accurate 42.3% of the time.	H High attrition rate; significant difference in the retention rate between the two treatment groups.	L
Sheehan *et al.*, 2009 [29]	U Not reported.	U	U	U	U	H High attrition rate.	L
Sheehan *et al.*, 2013 [30]	U Not reported.	L Study medications were encapsulated using a double-dummy design.	L	U	U	L	L
**Authors, Year**	**Sampling Bias**		**Other Bias**				
	**Recruitment Strategies**	**Inclusion/Exclusion Criteria**	**Power Calculation**		**Adjunctive Medication**	**Adjunctive Bias**	
Goddard *et al.*, 2015 [26]	H Mixed recruitment strategies	H Mixed criteria to define SSRI/SNRI resistance (inclusion criterion).	Power calculation performed. Study powered to detect large effect sizes. H for small-to-moderate effects.		L Not permitted; urine toxicology performed to detect surreptitious use of benzodiazepines.		
Prosser *et al.*, 2009 [27]	H Mixed recruitment strategies	H Sample with mixed diagnoses (no separate analyses in the PD subgroup). Lack of specific panic symptom measures as inclusion criteria.	U No power calculation reported.		U Not permitted; urine toxicology not declared.	U for previous medications: period of withdrawal not reported. H for medication initiation: no titration in one of the two treatment groups (paroxetine); H for baseline imbalance: higher baseline depression severity in one of the two groups (paroxetine), related to outcome measures.	
Suppes *et al.*, 2014 [28]	U	H Sample with mixed diagnoses (no separate analyses in the PD subgroup). Lack of specific panic symptom measures as inclusion criteria.	Power calculation performed. Study powered to detect large effect sizes. H for small-to-moderate effects.		L Some adjunctive medications permitted. No differences in adjunctive medication distribution between the two treatment groups.		
Sheehan *et al.*, 2009 [29]	L	H Sample with mixed diagnoses (no separate analyses on panic symptoms in the PD subgroup). Lack of specific panic symptom measures as inclusion criteria.	U No power calculation reported.		L Some adjunctive medications permitted. No significant influence on the results was found.	H for baseline imbalance: higher rate of participants with mixed mood state and with PD in one of the two groups (risperidone).	
Sheehan *et al.*, 2013 [30]	U	H Lack of specific panic symptom measures as inclusion criteria.	U No power calculation reported.		L Some adjunctive medications permitted. No differences in adjunctive medication distribution between groups.		

L = low risk of bias; H = high risk of bias; U = unclear: insufficient information to permit judgment of low or high risk (for instance, the generation process of a randomized sequence was not specified); PD = panic disorder; PLB = placebo; SNRI = selective-norepinephrine reuptake inhibitor; SSRI = selective-serotonin reuptake inhibitor.
